# Household Hints and Recipes

**Published:** 1874-04

**Authors:** 


### Household Hints & Recipes.

To protect delicate drawings in pencil or chalk, such as are easily smudged if roughly handled, and to give them more permanence and solidity, it is well to coat them with collodion. The same may, if desired, be used with an admixture of paraffine, stearine, or castor oil, and affords them an excellent coating. Pencil sketches are in this way rendered clearer, and may therefore be copied the more easily when so treated.—*The Lens.*

---

CAUSTICS VS. WEEDS.—A recent writer, of a chemical turn of mind, says: "Two years ago I took a large house and grounds which had been uninhabited and utterly neglected for three years. The lawn is nearly an acre in extent. Dandelions, buttercups, plantains, docks, etc., were in the ascendant. After many attempts to eradicate them, I found at last that one drop of the common strong sulphuric acid dropped upon the crown of each weed entirely destroyed it, and it will never grow again. I used one of the ribbed bottles employed by chemists and photographers for dropping poisons, and found it to answer perfectly, and it enables one to apply the acid with great rapidity. Large docks, which have hitherto never been destroyed except by digging up, are effectually destroyed by the acid."

---

WATERPROOFING BOOTS.—Paraffine is recommended for this purpose in an English journal. The writer says: "Melt thoroughly the paraffine, and, having well warmed the boot, apply the paraffine with a brush or piece of flannel before a fire, to allow of the leather absorbing the liquid. I have tried the above, and it answers admirably, resisting snow-water during a week's shooting."

---

INFANTILE THUMB SUCKING NOT A HARMLESS HABIT.—Dr. Horace Dobell, in the *British Medical Journal*, remarks: "I have observed that a peculiar and rather common deformity of the chest is caused by the habit of sucking the thumb in infancy and early childhood. The weight of the arm on the thorax of the child during sleep produces depression of the ribs in the line occupied by the arm when the thumb is placed in the mouth. As this is a very important effect of 'thumb-sucking' never hitherto pointed out, I think it desirable to place this note on record for the benefit of other observers."

---

HAY-ASTHMA.—A correspondent of the *Scientific American*, who designates himself as an M. D., says that a decoction of poppy-heads taken from the garden, and made in a common tea-kettle, and the vapor inhaled, will afford relief from this most distressing malady. If the poppy-heads cannot be had, then half a teaspoonful of laudanum may be added to a pint of water, and the steam inhaled in the same way. Of course poppy-capsules, which can be obtained in any drug store, may take the place of the heads, as recommended.

---

DIABETIC BREAD.—In a somewhat recent number of the *Bulletin General de Therapeutique*, Mr. Dannecy proposes the use of bread made from roasted flour for diabetic patients, instead of gluten biscuit. He asserts that roasted starch cannot be converted into glucose, and that bread made out of the various farinas so torrefied is greedily eaten by patients who have been restricted to the ordinary preparation of gluten until they have become thoroughly disgusted. Moreover, under its use the thirst lessens, and the digestive derangements are markedly ameliorated.

---

PERPETUAL PASTE.—Dissolve a teaspoonful of alum in a quart of water. When cold, stir in as much flour as will give it the consistency of thick cream, being particular to beat up all the lumps; stir in as much powdered rosin as will lay on a dime, and throw in half a dozen cloves to give it a pleasant odor. Have on the fire a teacup of boiling water, pour the flour mixture into it, stirring well at the time. In a few minutes it will be of the consistency of mush. Pour it into an earthen or china vessel; let it cool; lay a cover on, and put in a cool place. When needed for use take out a portion and soften it with warm water. Paste thus made will last twelve months. It is better than gum, as it does not gloss the paper, and can be written on.

---

CURE FOR BALDNESS.—An English paper relates the following case:

A gentleman, who had lost nearly all his hair after a very severe attack of fever, consulted a French physician of great reputed success as a hair-restorer. The prescription was a drachm of the homœopathic tincture of phosphorus to one ounce of castor oil; the bare spot to be rubbed with this mixture three times weekly for half an hour each time, after the skin of the head had been thoroughly cleansed with warm water without soap. This treatment was faithfully carried out for about six months; the hair soon began to grow, and in a year from the time of first following the doctor's advice, his head was as thoroughly covered as ever, the new crop of hair being about two shades darker than the old.

---

EATING WHEN SICK.—It is the custom among a certain class of people, when a member of the family falls sick, to begin at once to ask, "Now what can you eat?" Every one has heard of the old story of the man who always ate eighteen apple dumplings when he was sick. On one occasion when he was engaged upon the eighteenth, his little son said, "Pa, give me a piece." "No, no, my son," replied the father, "go away; pa is sick." When a young man has surfeited in season and out of season, until exhausted nature gives way, and a fever is coming on, the good mother is in trouble. She anxiously inquires, "Now, John, what can you eat? You must eat something! People can't live without food!" Then comes toast and tea, etc. The stomach is exhausted, and no more needs stimulating or food than a jaded horse needs the whip. What is needed is rest, complete rest. Nine-tenths of the acute diseases might be prevented by a few days' starvation when the first indications appear. I don't mean complete abstinence in every case, but perhaps a piece of coarse bread, with cold water for drink. If such a policy were generally adopted, what ruin would overtake the medical profession. How many physicians would lack for patients.

---

GREASE ERASER.—The following will be found to be a most excellent preparation for removing grease spots from clothing:

Benzine,
Alcohol,
Ether, of each, equal parts.

Apply with a sponge, lay a sheet of tissue-paper on the garment, and dry with a hot sad-iron. It does the work effectually, without the slightest injury to the most delicate fabric. It seems to be a perfect imitation of an article called "Etheriolene."

A CEMENT to stop cracks in glass vessels to resist moisture and heat:—Dissolve caseine in cold saturated solution of borax, and with this solution paste strips of hog's or bullock's bladder (softened in water) on the cracks of glass, and dry at a gentle heat; if the vessel is to be heated, coat the bladder on the outside, before it has become quite dry, with a paste of a rather concentrated solution of silicate of soda and quicklime or plaster-of-Paris.

---

WHEN fuel is burned in an open fire-place, at least seven-eighths of the actual or potential heat passes up the chimney unused; about one-half being carried off with the smoke, and one-fourth with the current which flows in between the mantel-piece and the fire, while the remaining loss is represented by the unburned carbonaceous matter in the smoke.

---

SUNSHINE AND SLEEP.—Sleepless persons should court the sun. The very worst soporific is laudanum, and the very best, sunshine. Therefore it is very plain that poor sleepers should pass as many hours as possible in the sunshine, and as few as possible in the shade. Many women are martyrs, and yet they do not know it. They shut the sunshine out of their houses and their hearts, they wear veils, they carry parasols, they do all possible things to keep off the most potent influence which is intended to give them strength, and beauty, and cheerfulness. Is it not time to change all this, and so get color and roses in our pale cheeks, strength in our weak backs, and courage in our timid souls? The women of America are pale and delicate, but with the aid of sunlight they may be blooming and strong.—*Home and Health.*

---

CORNS.—A mixture of equal parts of glycerine and carbolic acid, applied with a camel's-hair pencil, is recommended as an excellent remedy for corns.

---

FOUL ULCERS.—The value of roasted coffee as a disinfectant, or rather deoderizer, is well known to housewives, and in the London *Lancet,* Dr. William Story commends it highly as an application to foul ulcers.

---

FLIES ON HORSES.—According to a French journal, horses and other animals may be protected from the persecutions of flies by painting with a pencil the insides of the ears, or other parts liable to be bitten, with a few drops of empyreumatic juniper oil (*huile de cade.*)

